# C9orf16 represents the aberrant genetic programs and drives the progression of PDAC

**DOI:** 10.1186/s12885-022-10202-5

**Published:** 2022-10-28

**Authors:** Xiaojun Chen, Hong Zhang, Bo Xiao

**Affiliations:** Department of Immunology, Tianjin Key Laboratory of Cellular and Molecular Immunology, Key Laboratory of Immune Microenvironment and Disease of the Educational Ministry, Tianjin Medical University, 22 Qixiangtai Road, Heping District, Tianjin, 300070 China

**Keywords:** Pancreatic ductal adenocarcinoma (PDAC), Single cell RNA-sequencing (scRNA-seq), C9orf16, MYC, Metastatic

## Abstract

**Background:**

Pancreatic ductal adenocarcinoma (PDAC), constituting 90% of pancreatic cancers, is the fourth leading cause of cancer-related deaths in the world. Lack of early detection of PDAC contributes to its poor prognosis as patients are often diagnosed at an advanced stage of disease. This is mostly due to the lack of promising diagnostic and therapeutic targets and corresponding drugs.

**Methods and results:**

Here, by bioinformatic analysis of single cell RNA-sequencing data on normal pancreas tissues, primary and metastatic PDAC tumors, we identified a promising PDAC biomarker, C9orf16. The expression of C9orf16, rarely detectable in normal epithelial cells, was upregulated in primary PDAC cancer cells and was further elevated in metastatic PDAC cancer cells. Gain or loss of function of C9orf16 demonstrated its critical functions in regulating the cell proliferation, invasion and chemotherapy resistance of cancer cells. Pathway analysis and functional studies identified MYC signaling pathways as the most activated pathways in regulating C9orf16 expression and in mediating the development and progression of PDAC.

**Conclusions:**

These data suggested a crucial gene regulation system, MYC-C9orf16, which is actively involved in PDAC development and progression, and targeting this system should be a novel diagnostic and therapeutic target for PDAC.

**Supplementary Information:**

The online version contains supplementary material available at 10.1186/s12885-022-10202-5.

## Introduction

Pancreatic ductal adenocarcinoma (PDAC) is known as the most common type of pancreatic cancers [1]. The 5-year overall survival rate of PDAC has increased from 3% in the 1970s to about 9% in 2020 [2]. The modest improvement of PDAC in survival rate is much lower than that in many other tumor types, and this fact makes PDAC a major medical challenge in malignancy therapies [3]. In the past few years, surgical treatment and adjuvant chemotherapy may prolong the survival of PDAC patients, but the prognosis of PDAC patients remains poor mostly due to the lack of early diagnostic methods and accurate recurrence and prognostic biomarkers [4–6]. Although many researchers are developing strategies to target oncogenes of PDAC, most of the results are unsatisfactory in clinical practice [7].

Recent years, increasing strategies are under development to target potential biomarkers by multiple methods, including simultaneously inhibiting multiple molecules or pathways, modification of mutant residues by small molecules, and RNA interference [7]. Thanks to the development of advanced technologies and increasing amount of bioinformatic information, aberrant genetic events in PDAC are generally demonstrated, and many ectopic genes were observed in PDAC, including KRAS, TP53, CDKN2A and SMAD4 [8]. Aberrant expression or mutation of these genes have been detected in early-stage intraepithelial neoplasia and are required for PDAC development [9–11]. In addition to the above most involved genes in PDAC, genes associated with stabilizing chromatin, remodeling chromatin, or editing point mutations in cancer cells, including BRCA, APOBEC and KDM6A, are also identified in PDAC [7, 12, 13]. Many cancer related signaling pathways, including TGF-β-SMAD4 [14], RAS-RAF [15], EGFR [16], P53 [17] and epigenetic pathways [18, 19], have been shown to be dysregulated in either human PDAC tissues or related animal models. Although the studies on these targets and pathways have achieved significant advances in understanding the tumorigenesis of PDAC, many are unsatisfactory in clinical and preclinical trials of therapies, and further investigations are urgently warranted.

In this study we identified a novel target gene, C9orf16, phenotypically and functionally study of which revealed the potential of this gene as an early biomarker and therapeutic target in PDAC research and clinical invention. C9orf16 (Chromosome 9 open reading frame 16) is a protein encoding gene, the functions of which were largely unknown. Reports on C9orf16, although most are briefly involved, in human are mostly related to cancer formation and invasion, suggesting a potential role of this gene in cancer development and progression [20–22]. Pathway analysis revealed several MYC related pathways as the core regulators of C9orf16 expression and PDAC development. Functional studies on PDAC cancer cell lines demonstrated some critical functions of MYC-C9orf16 in regulating cancer cell migration, invasion and chemotherapy-induced apoptosis. These findings suggested that the novel gene axis, MYC-C9orf16, might play some critical function in regulating PDAC progression and further investigation on this target might provide a novel research or even therapeutic target for PDAC.

## Materials and methods

### Ethics statement

This study has been approved by the Institutional Review Board (IRB) of Tianjin Medical University. All experiments in this study were approved by the Institutional Review Board of Tianjin Medical University. All the methods used in this study were carried out in accordance with the guidelines and regulations outlined by the Institutional Review Board and Ethical Committee of Tianjin Medical University. The data used in this were publicly available and informed consent is not required for data acquisition. No humans or tissue samples taken from humans were involved in this study. Informed consent is not applicable.

### Bioinformatics analysis

Three single cell RNA-sequencing (scRNA-seq) datasets were downloaded from the Gene Expression Omnibus (GEO): GSE85241, normal human pancreases (human donors) [23]; GSE141017, primary human PDAC (surgical resection) [24]; GSE154778, primary (surgical resections) and metastatic (biopsies of 6 patients: 5 liver metastases and 1 omentum metastasis) PDAC [25]. The matrix data were read and processed by Seurat package in R-studio (Version 1.4.1717) [26] and three datasets were integrated [27]. Cell clusters were identified and differentially expressed genes were evaluated. Gene expression was visualized by Uniform Manifold Approximation and Projection (UMAP), violin plots or dot plots. Differential expression analysis was done by “FindAllMarkers” function and heatmaps were generated by “DoHeatmap” function in Seurat package R-studio (https://satijalab.org/seurat/articles/pbmc3k_tutorial.html). For signaling pathway analysis, differentially expressed genes (with the fold change and p-value of each gene) were loaded into Ingenuity Pathway Analysis [28] for the upstream regulator and pathway identification.

### Cell line culture and storage

All the cell lines used in this study, including cancer cell lines from human pancreatic tumors, AsPC-1, SW1990, BxPC-3, Capan-1, Hs 766 T and PANC-1, and the human normal pancreatic ductal epithelial cell line (HPNE), were purchased from American Type Culture Collection (ATCC) within the past ten years. All the cells were confirmed to be free of mycoplasma contamination. The cells were cultured in 5% CO2, 37 °C, saturated humidity in cell culture medium supplied with 10% fetal bovine serum (FBS) and the antibiotics Penicillin (100 IU) and Streptomycin (100 µg/ml) and stored in liquid nitrogen in cell frozen medium (Medium: FBS = 1:1).

### Immunohistochemistry analysis

Immunohistochemical staining was performed on the human tumor and benign slides from the same patients with PDAC. Briefly, the slides were deparaffined and rinsed, endogenous peroxidases were inactivated by 3% hydrogen peroxide and antigen retrieval was performed by heating the slides in citrate buffer pH 6.0. The primary antibody used was C9orf16 antibody (Sigma-Aldrich, HPA020725, 1:500) and secondary antibody was goat anti-rabbit IgG H&L (HRP) (abcam, ab6721. 1:1000). DAB substrate kit (abcam, ab64238) was used to visualize the staining according to manufacturers’ instructions. C9orf16 protein immune-expression was evaluated by counting the number of stained cells in over 500 tumor cells in five different tumor/ benign fields from five PDAC patients.

### RT-PCR and real time PCR analysis

After treatment, cell medium was aspirated, and the cells were washed by PBS. RNA was isolated using RNeasy Mini Kit from QIAGEN (74,104) from the cells according to manufacturer’s instructions. The purity and concentration of RNA detected by a NanoDrop™ 2000. High-Capacity cDNA Reverse Transcription Kit was used for reverse transcription (ThermoFisher Scientific, Catalog number: 4374967). The gene expression was determined by either recombinant Taq DNA Polymerase (ThermoFisher Scientific, Catalog number: EP0401) or SYBR™ Green PCR Master Mix (ThermoFisher Scientific, Catalog number: 4309155). For RT-PCR, the PCR product was run on agarose gel electrophoresis and the gel was imaged for analysis. For real time PCR analysis, the relative gene expression levels of gene were determined using the 2-ΔΔCt method. Human GAPDH was used as internal control gene for both assays. The primers were ordered from ORIGENE (C9orf16 Human qPCR Primer Pair (NM_024112), Catalog number: HP214755, sequences: forward, 5’- CAACTCCATGCTGGACCAGATC—3’, reverse, 5’- CTGCTGGAACTCCAGGCGTGT—3’; GAPDH Human qPCR Primer Pair (NM_002046), Catalog number: HP205798, sequences: forward, 5’- GTCTCCTCTGACTTCAACAGCG—3’, reverse, 5’- ACCACCCTGTTGCTGTAGCCAA—3’).

### C9orf16 knockdown and activation assays

C9orf16 knockdown was performed in PANC-1 cells and BxPC-3 cells, and activation was performed in HPNE cells via lentivirus infection. The C9orf16 shRNA(h) Lentiviral Particles and C9orf16 Lentiviral Activation Particles(h) were ordered from Santa Cruz Biotechnology (C9orf16 shRNA (h) Lentiviral Particles, Catalog number: sc-92859-V, C9orf16 Lentiviral Activation Particles (h), Catalog number: sc-413133-LAC). 48 h after lentivirus infection, the C9orf16 knockdown or activation cells were selected by puromycin treatment (Santa Cruz Biotechnology, Catalog number: sc-108071) and the cells were maintained in puromycin containing medium. The knockdown and activation efficiencies were confirmed by real time PCR or western blotting (see below).

### Western blotting analysis

The cells were harvested by trypsin and rinsed by PBS and lysed by RIPA Lysis Buffer (ThermoFisher Scientific, Catalog number: 89900). Equal amounts of protein (20–80 μg) from each sample were fractionated using sodium dodecyl sulphate–polyacrylamide gel electrophoresis (SDS-PAGE) and then transferred to polyvinylidene fluoride (PVDF) membranes. The PVDF membrane was then incubated with the indicated rabbit anti-C9orf16 antibody primary (Novus Biological, Catalog number: NBP1-83,955) and then goat anti-rabbit IgG (H + L) secondary antibody, HRP (ThermoFisher Scientific, Catalog number: 31460). The membrane was then developed by ECL western blotting substrate (ThermoFisher Scientific, Catalog number: 32106). GAPDH was used as internal control (rabbit anti-FAPDH antibody primary, Novus Biological, Catalog number: NB100-56,875).

### Cell proliferation analysis

Cell proliferation of the cell lines was assessed by MTT assays. In brief, cells with C9orf16 knockdown or overexpression and the corresponding control cells were plated into 96-well plates at 1.0 × 10^4^ cells/well and eight repeats were included for each condition. Culture medium was replaced with fresh medium containing 0.5 mg/ml MTT and cells were incubated for 3 h at 37 °C in the cell culture incubator. The medium was removed, and the cells were washed twice with PBS. Then 100 μl of DMSO was added to each well and the plates were analyzed by measuring the optical density at 570 nm.

### Cancer cell migration and invasion assay

5 × 10^4^ cancer cells from each condition were loaded into the transwell chambers for migration assay and the transwell chambers were precoated with Matrigel for invasion assay. The cells were cultured for 24 h and the cell on the upper surface of the membrane were removed and the cells on the bottom were stained with Crystal violet solution (Sigma-Aldrich, Catalog number: V5265). The membrane was dried and imaged. For quantification analysis, cells on three random regions of each membrane were counted manually and each experiment was independently repeated for at least three times.

### Cisplatin induced cell apoptosis

Cell apoptosis was induced by cisplatin, one of the most potent antitumor agents that activate several signal transduction pathways to induce cancer cell apoptosis [29] and has been used as a mainstay of chemotherapeutic agents in combination with other drugs or radiotherapy for PDAC therapy [30–34]. After incubating the cells with 20 μM cisplatin for 24 h, the cells were stained with APC Annexin V (ThermoFisher Scientific, Catalog number: A35110) and dead cell marker (ThermoFisher Scientific, Catalog number: 65–0865-14) and analyzed by a BD Arial II cell analyzer. The cells were divided into four quadrants (Q1 – Q4) and percentage of late apoptotic (Q2) cells were quantified.

### Statistical analysis

All experiments were repeated at least three times and all results were presented as mean ± SEM. All statistical data were analyzed and visualized by GraphPad Prism 8.0. Student’s two-tailed t test or 2-way ANOVA were used for comparing difference significance between 2 or more groups. p value of < 0.05 was considered statistically significant. *, *p* < 0.05, **, *p* < 0.01, ***, *p* < 0.001, ****, *p* < 0.0001.

## Results

### Identification of gene profiles of primary and metastatic PDAC

Pancreatic ductal adenocarcinoma (PDAC) is a hostile solid malignancy coupled with an extremely high metastatic rate in most patients at the time of diagnosis [35]. To study the gene expression profiles and critical genes regulating the development and metastasis of PDAC, we searched the Gene Expression Omnibus (GEO) and found three scRNA-seq datasets on normal human pancreas [23], primary [24] and metastatic human PDAC [25], respectively. These data were read and integrated, and doublets/debris with extremely high/low gene and molecular numbers and cells with high percentage of mitochondria genes were removed (Fig. S1). The remaining cells were clustered and the distribution of cells from each sample (Fig. S2A), each dataset (Fig. S2B) and each disease status (Fig. S2C) in the integrated data was visualized by UMAPs. As the pancreatic and PDAC tumor tissues are composed of multiple cell types, the expression of different cell type specific marker genes was checked [23–25] (Fig. S2D) and major cell type were identified based on these gene expression (Fig. S2E-Fig. S2F), including epithelial cell lineages (Epi/Can), fibroblasts (Fib), myeloid cells (Mye), T cells (T) and endothelial cells (endo). The cell type definition was confirmed by the expression specification of the cell type marker genes visualized by dot plots (Fig. S2G). Although the other cell types were mostly in primary tumor tissues, epithelial cell lineages were detectable in all tissue statuses (Fig. S2F). The gene expression patterns of each cell type were confirmed by heatmap of top 200 genes of each cell type (Fig. S2H).

**Fig. 1:**
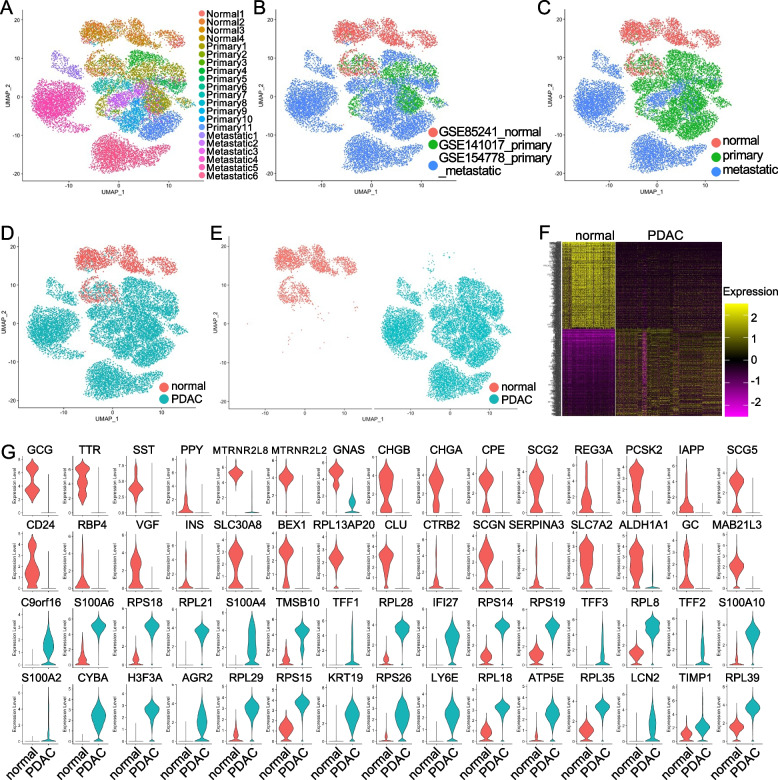
Epithelial cell lineage extraction from the integrated scRNA-seq data. **A-C** The epithelial cell lineages including normal epithelial and cancer cells were integrated and re-clustered, and visualization of each sample (**A**), each dataset (**B**) and each cancer status (**C**) by UMAP. **D-E** Distribution of the normal epithelial and cancer cells in combined (**D**) and split (**E**) UPMAs. **F** Heatmap showed the top 500 differentially expressed gene between normal epithelial cells and PDAC cancer cells by “DoHeatmap” function in R-studio (Version 1.4.1717). **G** The expression of the top 30 specific genes of normal epithelial cell and cancer cells was shown by violin plots

We then extracted the epithelial cell lineages from the integrated data and visualized the distribution of cells from each sample (Fig. 1A), each dataset (Fig. 1B) and each disease status (Fig. 1C) by UMAPs. Distinct distribution of cell clusters from different cancer status suggested that these epithelial/cancer cells showed different gene expression profiles. The purity of the epithelial lineages was confirmed by examining the expression of cell type marker genes (Fig. S3). The normal epithelial cells from the normal pancreatic tissues and cancer cells from primary and metastatic tumors were visualized (Fig. 1D-Fig. 1E) and the differentially expressed genes among normal epithelial and cancer cells were evaluated and visualized by heatmap of top 1,000 genes (Fig. 1F). The expression of top 30 genes in both normal epithelial cells and cancer cells were illustrated by violin plots (Fig. 1G). Notably, most of the genes specific to normal epithelial cells were related to normal functions of pancreatic epithelial cells, for example GCG, TTR, SST, PPY [36], MTRNR2L8 and MTRNR2L2 [37]. The most significantly upregulated gene that was specific to PDAC cancer cells was C9orf16, the functions of which in PDAC have never been reported. Other specific gene of PDAC included ribosome protein genes (Fig. 1G).

**Fig. 2:**
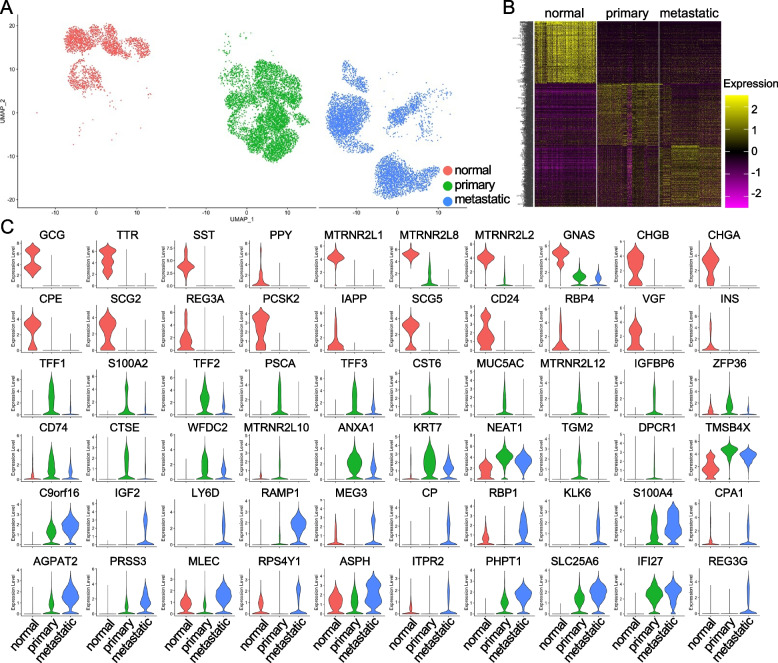
Differentially expressed genes of normal epithelial cells, primary and metastatic cancer cells. **A** Split UMAP of the normal epithelial cells, primary and metastatic cancer cells. **B** Heatmap of the expression of top 1000 genes of normal epithelial cells, primary and metastatic cancer cells by “DoHeatmap” function in R-studio (Version 1.4.1717). **C** Violin plots showed the expression of top 20 genes specific to normal epithelial cells, primary and metastatic cancer cells, respectively

The normal epithelial cells, primary and metastatic PDAC cancer cells were then separately visualized (Fig. 2A) and the differentially expressed genes among these three cell types were determined. Heatmap visualization of the expression of top 500 genes of each cell type suggested that these cells showed distinct gene expression profiles (Fig. 2B). We next showed the expression of top 20 genes of each cell type by violin plots (Fig. 2C). Similarly, most of the genes specifically expressed in normal epithelial cells were the pancreatic epithelial cell functional genes, and the primary cancer cells expressed the genes that were highly associated with the development of PDAC, including TFFs [38, 39], S100A2 [40], PSCA [41], CST6 [42] and many others. Again, the gene, C9orf16, was the one of the most significantly elevated genes in metastatic cancer cells compared to both normal epithelial cells and primary cancer cells (Fig. 2C), suggesting that specific expression of C9orf16 in PDAC cancer cells might be critical for the PDAC metastasis.

### C9orf16 expression was closely correlated with development and progression of PDAC

**Fig. 3:**
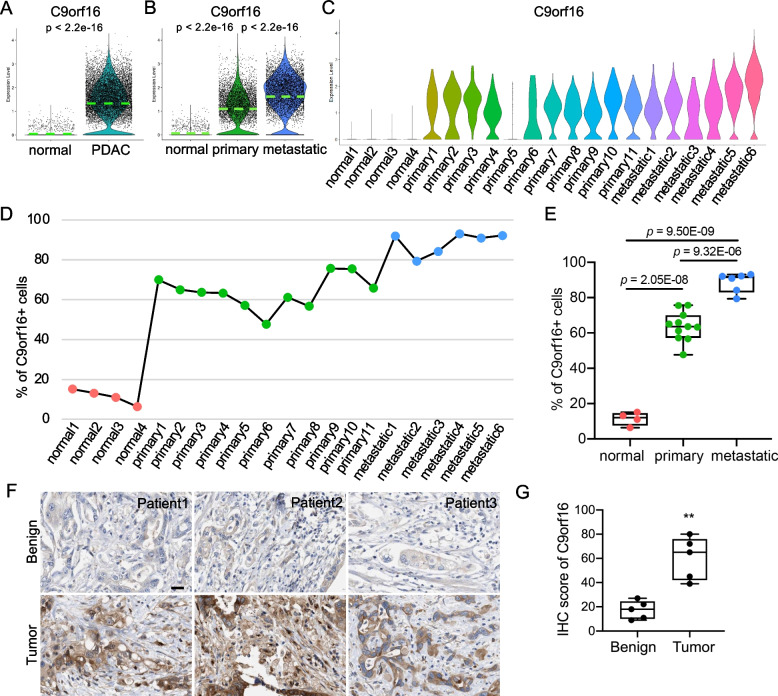
C9orf16 expression was associated with the development and progression of PDAC. **A-B** Violin plots showed comparison of C9orf16 expression in normal epithelial cells and PDAC cancer cells (**A**), normal epithelia cells, primary and metastatic cancer cells (**B**). Green dashed lines indicated the median expression levels of each cancer cell status. **C** C9orf16 expression in the epithelial and cancer cells of each sample. **D** Curve of the percentage of C9orf16 + cells in each sample along with the progression of PDAC. **E** Quantification of percentage of C9orf16 + cells in each cancer status. **F** Immunohistochemistry (IHC) of C9orf16 on paraffin slides of human PDAC tumors and benign tissues. **G** Quantification of the IHC scores of C9orf16 on paraffin slides of human PDAC tumors and benign tissues. **: *p* < 0.01. Scale bar: 20 μm

To better examine the expression of C9orf16, we isolated and double checked the expression of C9orf16 in normal epithelial cells and PDAC cancer cells by distinct violin plots. C9orf16 was barely detectable in normal epithelial cells but was significantly elevated in PDAC cancer cells (Fig. 3A). When we split the primary and metastatic cancer cells, C9orf16 was upregulated in both primary and metastatic cancer cells compared to normal epithelial cells (Fig. 3B) and its transcription level was even higher in metastatic cancer cells when compared to primary cancer cells (Fig. 3B). For its expression in each sample, C9orf16 was almost nondetectable in any normal samples, but the expression levels were dramatically increased in all the primary cancer cells and the expression levels were further upregulated in metastatic cancer cells (Fig. 3C). For quantification, the percentages of C9orf16 + cells in each sample were determined and the percentages increased along with the development and progression of PDAC in human (Fig. 3D and Fig. 3E). To verify the observations on these bioinformatic analyses, we accessed the IHC staining of C9orf16 on benign and tumor slides from patients with PDAC and PDAC tumors showed significantly elevated C9orf16 protein levels (Fig. 3F and Fig. 3G). These data suggested that C9orf16 expression was associated with and might be critical for the progression of PDAC in human.

### C9orf16 deficiency decreased cancer cell invasion and increased apoptosis

**Fig. 4:**
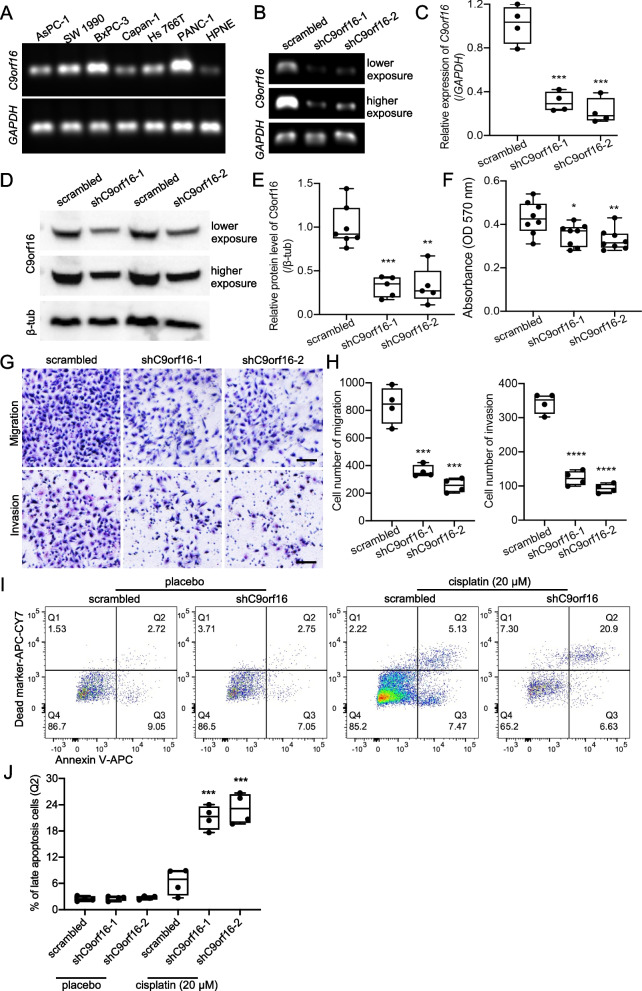
C9orf16 knockdown inhibited cancer cell invasion and increased apoptosis. **A** Expression of C9orf16 in pancreatic cancer cell lines and normal epithelial cells was examined by RT-PCR. **B** RT-PCR examination of C9orf16 knockdown cells and the scrambled cells showed the C9orf16 knockdown efficiency. **C** Real time PCR was used to check the C9orf16 knockdown efficiency. **D-E** Western blotting was performed to confirm the C9orf16 knockdown efficiency. **F** Cell proliferation measured by MTT assay showed decreased cell proliferation in PANC-1 cells with oeC9orf16 knockdown. Migration and invasion assays (**G**) and cell number quantification (**H**) of the C9orf16 knockdown and the scrambled cells. Flow cytometry analysis (**I**) of Cisplatin induced cell apoptosis and quantification of late apoptotic cell percentage (**J**) of the C9orf16 knockdown and the scrambled cells. Scale bar: 100 µm. **: *p* < 0.01; ***: *p* < 0.001; ****: *p* < 0.0001

To confirm the expression of C9orf16 in PDAC cancer cells, we examined the mRNA levels of C9orf16 in commonly used PDAC cancer cell lines and a normal pancreatic duct epithelial cell line, HPNE. Notably, HNPE cells showed lowest transcription of C9orf16, while the highly invasive PDAC cancer cell line, PANC-1, demonstrated the highest expression level of C9orf16 (Fig. 4A). To study the function of C9orf16 in PDAC cancer cell, we applied gene knockdown assay in PANC-1 cells by lentivirus infection. The mRNA levels and protein levels of the knockdown assay were confirmed by RT-PCR (Fig. 4B), real time PCR (Fig. 4C) and western blotting assays (Fig. 4D-Fig. 4E), respectively. C9orf16 knockdown significantly decreased PANC-1 cell proliferation (Fig. 4F). Cell migration and invasion are commonly used methods to check the ability of a cancer cell to change position within the tissues to undergo metastasis [43]. To check the phenotypical changes of the cancer cells after C9orf16 knockdown, we applied migration and invasion assays to the cells. Right to our expectation, PANC-1 cells showed significantly decreased cell migration and invasion after C9orf16 knockdown (Fig. 4G-Fig. 4H). Cisplatin is a very effective cancer drug for chemotherapy [44] and has had a major clinical impact in cancer therapy, including the treatment in patients with pancreatic cancers [45], by inducing cancer cell apoptosis and DNA damage. To study the effect of C9orf16 knockdown in cancer cell apoptosis, the Cisplatin treated cells were stained with Annexin V for flow cytometry. Clearly, C9orf16 deficiency significantly increased Cisplatin induced cancer cell apoptosis (Fig. 4I-Fig. 4J). To check the generalizability of C9orf16 functions, the gene knockdown assay was also applied in another PDAC cancer cell line with high C9orf16 expression, BxPC-3 (Fig. S4A-Fig. S4D). C9orf16 knockdown in BxPC-3 cells also caused remarkable decrease in cell migration and invasion (Fig. S4E and Fig. S4F).

### C9orf16 activation increased cell invasion and increased anti-apoptosis capacity

**Fig. 5:**
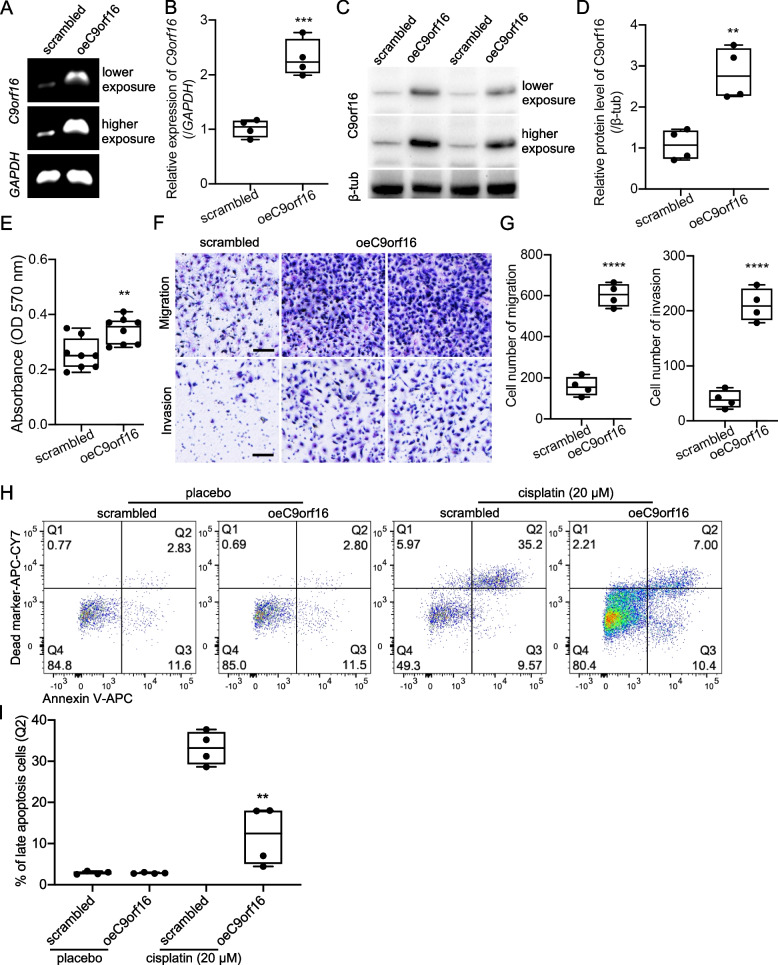
C9orf16 overexpression in normal pancreatic epithelial cell increases cell invasion and decreased apoptosis. **A-D** RT-PCR (**A**), real time PCR (**B**) and western blotting (**C-D**) analysis were performed to confirm the overexpression efficiency. **E** Cell proliferation measured by MTT assay showed increased cell proliferation in HPNE cells with oeC9orf16 overexpression. Migration and invasion assays (**F**) and cell number quantification (**G**) of the C9orf16 overexpression and the scrambled cells. Flow cytometry analysis (**H**) of Cisplatin induced cell apoptosis and quantification of late apoptotic cell percentage (**I**) of the C9orf16 overexpression and the scrambled cells. Scale bar: 100 µm. **: *p* < 0.01; ***: *p* < 0.001; ****: *p* < 0.0001

As normal pancreatic duct epithelial cell line, HPNE, showed little C9orf16 transcription, to further confirm the functions of C9orf16 in normal epithelial cells, we performed C9orf16 overexpression assay on HPNE. RT-PCR (Fig. 5A), real time PCR (Fig. 5B) and western blotting (Fig. 5C-Fig. 5D) confirmed significantly increased mRNA levels and protein levels in the overexpression line. Functionally, C9orf16 overexpression remarkably promoted normal epithelial cell proliferation (Fig. 5E), migration and invasion (Fig. 5F-Fig. 5G), suggesting that C9orf16 was required to facilitate the cells with these phenotypes. On the other hand, C9orf16 activation significantly decreased Cisplatin induced HPNE apoptosis (Fig. 5H-Fig. 5I). These data revealed that C9orf16 was required for the malignant phenotypes of pancreatic epithelial cells.

### C9orf16 deficiency decreased EMT in cancer cells

**Fig. 6:**
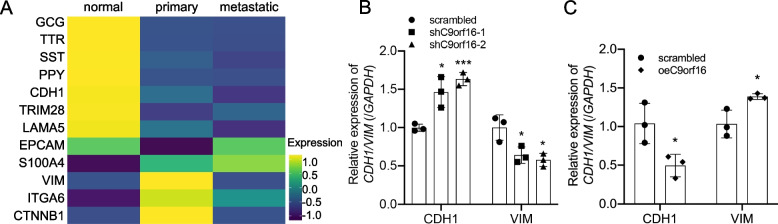
Activated EMT in PDAC cancer cells. **A** Heatmap visualization of the expression of epithelial cell marker genes and mesenchymal signature genes in normal epithelial cells, primary and metastatic cancer cells by “DoHeatmap” function in R-studio (Version 1.4.1717). Relative expression of the epithelial cell gene, CDH1, and mesenchymal cell gene, VIM, in C9orf16 knockdown cancer cells (**B**) and C9orf16 overexpression normal epithelial cells (**C**). *: *p* < 0.05; **: *p* < 0.01; ***: *p* < 0.001

Epithelial-mesenchymal transition (EMT) is a morphologic cellular program simply defined as the phenotypic transition from an epithelial to a mesenchymal state, which has been commonly reported in pancreatic cancers and considered the crucial event of carcinoma invasion and dissemination [46, 47]. As some mesenchymal signature genes, for example S100A4, were upregulated in cancer cell in the scRNA-seq analysis (Fig. 1G and Fig. 2C), we assumed that the cancer cells underwent activate EMT. To confirm that, we checked and visualized the expression of normal pancreatic epithelial cell marker genes, including GCG, TTR, SST, PPY [23], common epithelial cell markers, including CDH1, TRIM28, LAMA5 and EPCAM [48, 49], and mesenchymal signature genes, including S100A4, VIM, ITGA6 and CTNNB1 [49] in the normal epithelial cell and primary and metastatic cancer cells by heatmap (Fig. 6A, Fig. S5). We found that the epithelial cell marker genes were highly expressed in normal epithelial cells, while the mesenchymal signature genes were significantly upregulated in primary cancer cells and were roughly downregulated in metastatic cancer cells. These observations revealed that the EMT was significantly activated in primary cancer cells but stayed moderate in late metastatic cancer cells. To check the function of C9orf16 in cancer cell EMT, we examined the expression levels of CDH1 and VIM and found that C9orf16 deficiency dramatically increased the CDH1 mRNA levels but decreased VIM expression in PANC-1 cells, while C9orf16 overexpression significantly decreased CDH1 expression but increased the VIM mRNA levels (Fig. 6B-Fig. 6C). These data suggested that C9orf16 was required for the EMT of the cancer cells.

### Activated MYC pathways in PDAC cancer cells

**Fig. 7:**
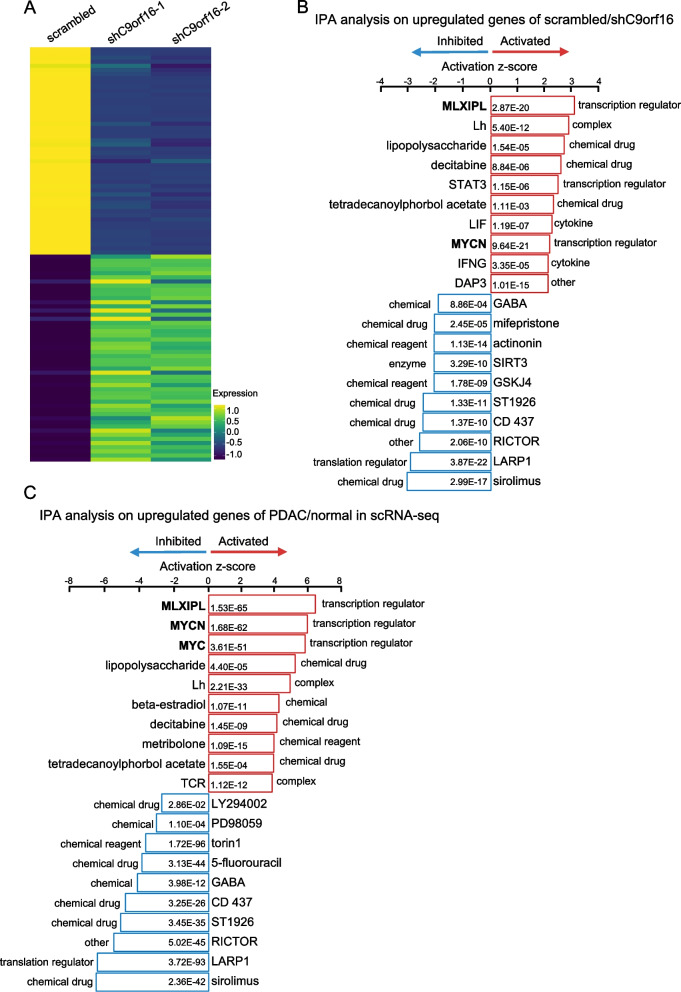
Pathway analysis revealed MYC signaling pathways as the top activated pathways. **A** Heatmap of the expression of the differentially expressed genes between C9orf16 knockdown and scrambled cancer cells by “DoHeatmap” function in R-studio (Version 1.4.1717). **B** Pathway analysis on differentially expressed genes of C9orf16 expression (scrambled) cancer cells relative to C9orf16 knockdown cells. **C** Pathway analysis on the differentially expressed genes of PDAC cancer cells relative to normal epithelial cells in the scRNA-seq data

To check the gene expression patterns of PANC-1 cells after C9orf16 knockdown, we performed RNA-sequencing on the C9orf16 knockdown cells and scrambled cells. The C9orf16 knockdown lines showed distinct genes expression profiles with the scrambled cells (Fig. 7A). Then we performed signaling pathway analysis by Ingenuity Pathway Analysis [50] on the cells and identified the top regulators of the C9orf16 expression (scrambled) lines. We found that among the top regulators, MYC related signaling pathways, including MLXIPL and MYCN, were the most significant ones (Fig. 7B), suggesting that MYC related signaling pathways might paly critical roles in regulating C9orf16 expression and potentially PDAC development and progression. To further confirm this, we then did IPA analysis on the differentially expressed genes of primary and metastatic cancer cells relative to normal epithelial cells in the scRNA-seq data. Surprisingly, we found that the top three most significant regulators were MLXIPL, MYC and MYCN (Fig. 7C), all were MYC related signaling pathways. These data further confirmed that the MYC related signaling pathways were significantly activated in C9orf16 expression cancer cells and in human in vivo cancer cells and might be the key regulators of PDAC development and progression.

### Blocking MYC signaling rescued cell invasion and promoted apoptosis

**Fig. 8:**
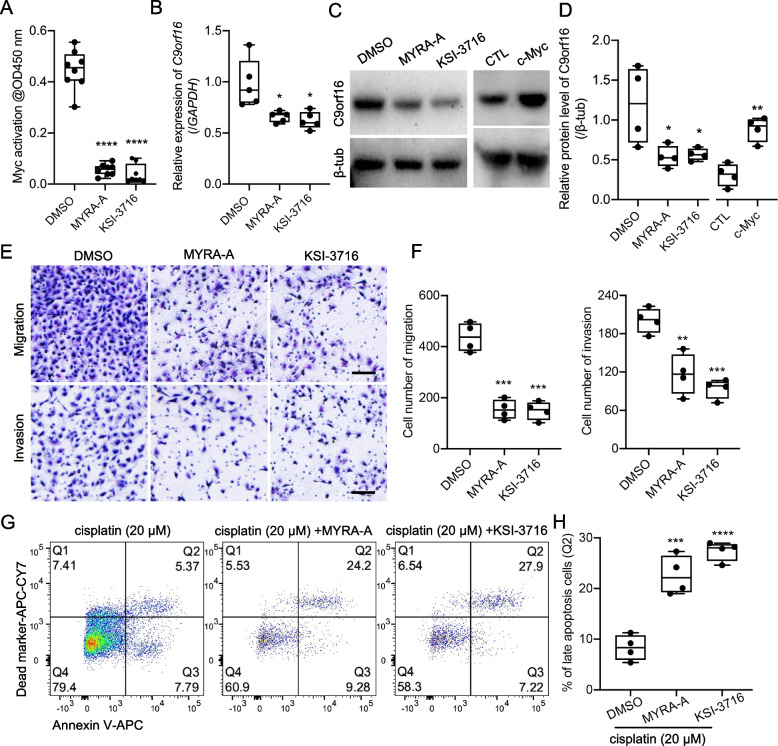
Blocking MYC signaling reduced cancer cell invasion and increased apoptosis. A MYRA-A and KSI-3716 treatment effectively blocked the activity of Myc signaling pathway in PANC-1 cells. **B** Real time PCR analysis of C9orf16 expression after MYRA-A and KSI-3716 treatment. **C**-**D** Western blotting analysis were performed to examine the expression of C9orf16 after the treatment of MYRA-A and KSI-3716 as well as recombinant human c-Myc protein. Migration and invasion assays (**E**) and cell number quantification (**F**) on cancer cells after MYRA-A and KSI-3716 treatment. Flow cytometry analysis (**G**) of Cisplatin induced cell apoptosis and percentage quantification of late apoptotic cells (**H**) of cancer cells after MYRA-A and KSI-3716 treatment. Scale bar: 100 µm. *: *p* < 0.05; **: *p* < 0.01; ***: *p* < 0.001; ****: *p* < 0.0001

As MYC signaling is necessary for the expression of C9orf16 and cancer development, we then attempted to block MYC signaling pathway by two commonly used small molecular inhibitors, MYRA-A and KSI-3716 [51, 52]. Treatment of PANC-1 cells by MYRA-A and KSI-3716 effectively blocked the MYC signaling (Fig. 8A) and significantly downregulated the expression of C9orf16 at both mRNA (Fig. 8B) and protein levels (Fig. 8C-Fig. 8D), while adding recombinant human c-Myc protein to PANC-1 cells remarkably increased the protein level of C9orf16 (Fig. 8C-Fig. 8D). Functionally, the cell migration and invasion were inhibited by both MYRA-A and KSI-3716 treatment (Fig. 8E-Fig. 8F), suggesting that these two chemicals were potential effective drugs for the blockage of PDAC metastatic related phenotypes. Similarly, the Cisplatin induced cell apoptosis of PANC-1 cells were significantly increased (Fig. 8G-Fig. 8H). These data implied that MYC signaling was required to regulate C9orf16 gene expression and subsequentially PDAC development and progression.

## Discussion

Pancreatic ductal adenocarcinoma (PDAC) is the fifth most common cause of cancer deaths worldwide and represents the most lethal diseases of the common malignancies [2, 53, 54]. Although some cases of PDAC detection at early stages have been reported [55], PDAC is typically diagnosed at an advanced stage (usually termed as advanced PDAC) [56]. Early detection of PDAC is very important to improve the prognosis of PDAC, but for PDAC patients with metastatic diseases (metastatic PDAC, PDAC with metastases in other organs) or with locally advanced diseases (advanced PDAC) not amenable to surgery due to the lack of visible and distinctive symptoms and reliable biomarkers for early diagnosis as well as aggressive metastatic spread[57, 58]. The mortality and morbidity rates after surgery have been improved recent years, mostly due to increased care in specialist centers and multidisciplinary approaches to perioperative management [59, 60]. However, for patients with advanced PDAC, the survival time after diagnosis is still extremely low [61, 62], which emphasizes an urgent need for novel effective markers in early diagnosis and for the treatment of patients with advanced and metastatic PDAC [54]. FOLFIRINOX and gemcitabine are approved as a first-line treatment for advanced PDAC not amenable for surgery, both with well-established, largely equivalent regimens and survival benefit [56], however both therapies, as well as their combinations with other chemotherapies, fail to provide expected results, prolonging life expectancy only moderately [58].

The identification of biomarkers with adequate sensitivity and specificity that can help guide the diagnosis and treatment decisions in PDAC remain a clinically unmet need, although a lot of efforts have been made using genomics, transcriptomics, proteomics, metabonomics, and bioinformatics [63]. Carbohydrate antigen (CA) 19–9 is a type of antigen released by pancreatic cancer cells and it has been referred to as the only biomarker approved for clinical PDAC diagnosis. However, recent studies suggest that it is insufficient as an independent diagnostic marker as it has limited sensitivity and specificity in symptomatic patients and may lead to false-positive results and misdiagnoses [64, 65]. Carcinoembryonic antigen (CEA) is another commonly used blood-based tumor biomarkers in clinical practice [66, 67]. Although the specificity of CEA is similar to that of CA 19–9, the sensitivity is even poorer at identification of PDAC. CA 125, a mucin-like transmembrane glycoprotein, is found to have a superior predictive ability at predicting resectability compared other biomarkers including CA 19–9 [68, 69] but this needs further clinical confirmation and validation in patients with potentially resectable tumors and aberrantly high levels of CA 125.

Besides the above commonly reported biomarkers, a multitude of other biomarkers have been identified in PDAC in recent years. However, prospective studies are badly needed to rigorously investigate the clinical impact of incorporating these biomarkers into clinical decision [70]. This is making it urgent to discover and validate both novel and known biomarkers for early detection, diagnosis, prediction of prognosis [71]. In the current study, we identified a novel PDAC biomarker, C9orf16, the expression of which was attractively associated with the development and progression of PDAC. C9orf16 is a protein coding gene and its functions have been less determined in human diseases including cancers. The few studies of C9orf16 are mostly on its involvement in human cancer, including ovarian cancer [20], colorectal cancer [22], lung cancer [21], hepatocellular carcinoma [72] and pancreatic cancer [73]. Although most of these studies just briefly mentioned C9orf16 as one of the differentially expressed genes, they stressed the potentials of this gene in cancer development and progression. Expression levels of C9orf16 were actively modulating the proliferation and invasion of the PDAC cancer cells. Deficiency of C9orf16 in advanced PDAC cancer cell line significantly upregulated cell apoptosis induced by another chemotherapy drug, cisplatin. Cisplatin displays a broad spectrum of anticancer activity, and is estimated to be administered to 40–80% of all cancer patients undergoing chemotherapy [74]. Cisplatin is used as a mainstay of chemotherapeutic agents in combination with other drugs or radiotherapy for early, advanced or metastatic PDAC in several ongoing clinical trials [75]. In cancer studies, cisplatin is commonly used as a drug to induce cancer cell apoptosis and drug screening with respect to its functions in inducing DNA injury [76]. In normal pancreatic epithelial cells in our study, C9orf16 overexpression remarkably decreased cisplatin induced cell apoptosis. Our current study is the first to reveal the detailed functions of C9orf16 along with the progression of PDAC in human both in vivo and in vitro.

The oncogene c-MYC (MYC) is a well-known transcription factor that has been implicated in the pathogenesis of over thirty percent of all human malignant cancers [77]. MYC has been confirmed to induce relentless tumor growth associated with increased DNA replication and transcription, cellular proliferation and growth, protein synthesis and increased tumor cell metabolism [78]. PDAC is reported to be one particular cancer with both evidence for MYC as a central effector and only marginal therapeutic success [79]. Upregulation of MYC occurs in over 42% of advanced PDAC in human [80, 81], which has been remarkably demonstrating the putative oncogenic impact of this gene in PDAC. A most recent study shows that MYC promotes PDAC metastasis by recruiting tumor associated macrophages (TAMs), leading to greater bloodstream intravasation. Their data implicate MYC activity as a major determinant of metastatic burden in advanced PDAC [50]. Besides the deregulation of MYC itself, this transcriptional factor has been shown to regulate several genes that are highly associated with the oncogenesis of PDAC [82, 83]. Among these direct and undirect targets, in the current study, we first identified that MYC signaling pathways were upstream regulators of C9orf16 in PDAC and targeting MYC by inhibitors significantly downregulated the expression of C9orf16 and then the invasion of cancer cells. Although the detailed molecular mechanisms were undetermined, these data revealed a novel gene axis MYC-C9orf16 that was closely related to the development and progression of PDAC. Targeting this gene axis might shed light on the research value of C9orf16 in PDAC and might further propose a novel therapeutic target for the clinical treatment of PDAC.

## Supplementary Information

**Additional file 1:** **Supplemental Fig. S1.** Quality control of the single cell RNA-sequencing data. (A) Three single cell RNA-sequencing datasets were integrated, and the cell qualities were visualized by violin plots. (B) Low quality cells were removed based on gene number (nFeature_RNA), molecular number (nCount_RNA) and mitochondria gene percentage (percent.mt) of each cell. **Supplemental Fig. S2.** Integration of scRNA-seq data on primary pancreases, primary and metastatic PDAC tissues. Three single cell RNA-sequencing datasets were integrated, and visualization of each sample (A), each dataset (B) and each cancer status (C) by UMAP. (D) The expression of canonical cell type marker genes used for cell type identification. (E) Cell type definition based on the expression of cell type marker genes. (F) Split version of the cell type identification. (G) Dot plots showed the expression of the cell type marker genes in the defined cell types. (H) Heatmap of the expression of top 500 genes of each defined cell type in (E) was generated by “DoHeatmap” function in R-studio (Version 1.4.1717). **Supplemental Fig. S3.** Purity of the epithelial cell lineage extracted were confirmed by the expression of canonical cell type markers. **Supplemental Fig. S4.** C9orf16 knockdown in BxPC-3 cells inhibited cancer cell invasion. RT-PCR (A), real time PCR (B) and western blotting (C-D) analysis were performed to confirm the knockdown efficiency of C9orf16 in BxPC-3 cells. Migration and invasion assays (E) and cell number quantification (F) of the C9orf16 knockdown and the scrambled BxPC-3 cells. Scale bar: 100 µm. **: *p* < 0.01; ***: *p* < 0.001; ****: *p* < 0.0001. **Supplemental Fig. S5.** Violin plots showed the expression of EMT related genes. **Supplemental Fig. 6.** Original full-length gels and blots for each figure panel. **Supplemental Fig. 7.** Original full-length gels and blots for each panel of Supplemental Fig. 4.

## Data Availability

All data generated and analyzed during this study are included in this published article and its supplementary information files.
