# Supplementary material for: C9orf16 represents the aberrant genetic programs and drives the progression of PDAC
Source: BMC Cancer. 2022 Oct 28;22:1102. doi: 10.1186/s12885-022-10202-5 (PMC9615161; doi:10.1186/s12885-022-10202-5)
Supplement: Supplementary file 1 — Additional file 1: Supplemental Fig. S1. Quality control of the single cell RNA-sequencing data. (A) Three single cell RNA-sequencing datasets were integrated, and the cell qualities were visualized by violin plots. (B) Low quality cells were removed based on gene number (nFeature_RNA), molecular number (nCount_RNA) and mitochondria gene percentage (percent.mt) of each cell. Supplemental Fig. S2. Integration of scRNA-seq data on primary pancreases, primary and metastatic PDAC tissues. Three single cell RNA-sequencing datasets were integrated, and visualization of each sample (A), each dataset (B) and each cancer status (C) by UMAP. (D) The expression of canonical cell type marker genes used for cell type identification. (E) Cell type definition based on the expression of cell type marker genes. (F) Split version of the cell type identification. (G) Dot plots showed the expression of the cell type marker genes in the defined cell types. (H) Heatmap of the expression of top 500 genes of each defined cell type in (E) was generated by “DoHeatmap” function in R-studio (Version 1.4.1717). Supplemental Fig. S3. Purity of the epithelial cell lineage extracted were confirmed by the expression of canonical cell type markers. Supplemental Fig. S4. C9orf16 knockdown in BxPC-3 cells inhibited cancer cell invasion. RT-PCR (A), real time PCR (B) and western blotting (C-D) analysis were performed to confirm the knockdown efficiency of C9orf16 in BxPC-3 cells. Migration and invasion assays (E) and cell number quantification (F) of the C9orf16 knockdown and the scrambled BxPC-3 cells. Scale bar: 100 µm. **: p < 0.01; ***: p < 0.001; ****: p < 0.0001. Supplemental Fig. S5. Violin plots showed the expression of EMT related genes. Supplemental Fig. 6. Original full-length gels and blots for each figure panel. Supplemental Fig. 7. Original full-length gels and blots for each panel of Supplemental Fig. 4. [file 12885_2022_10202_MOESM1_ESM.pdf]

Figure S1

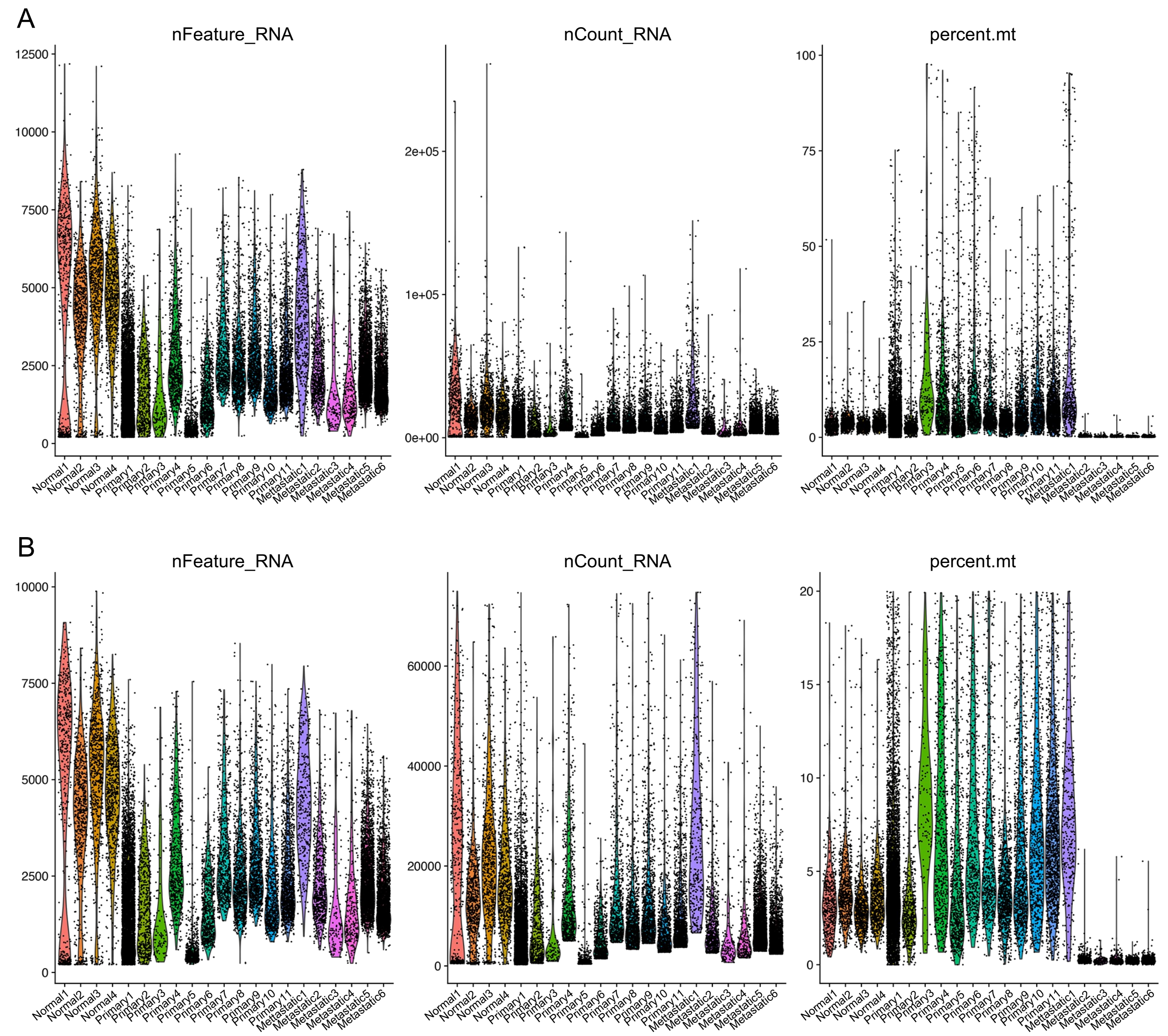

Supplemental Fig. 1 Quality control of the single cell RNA-sequencing data. (**A**) Three single cell RNA-sequencing datasets were integrated, and the cell qualities were visualized by violin plots. (**B**) Low quality cells were removed based on gene number (nFeature_RNA), molecular number (nCount_RNA) and mitochondria gene percentage (percent.mt) of each cell.

Figure S2

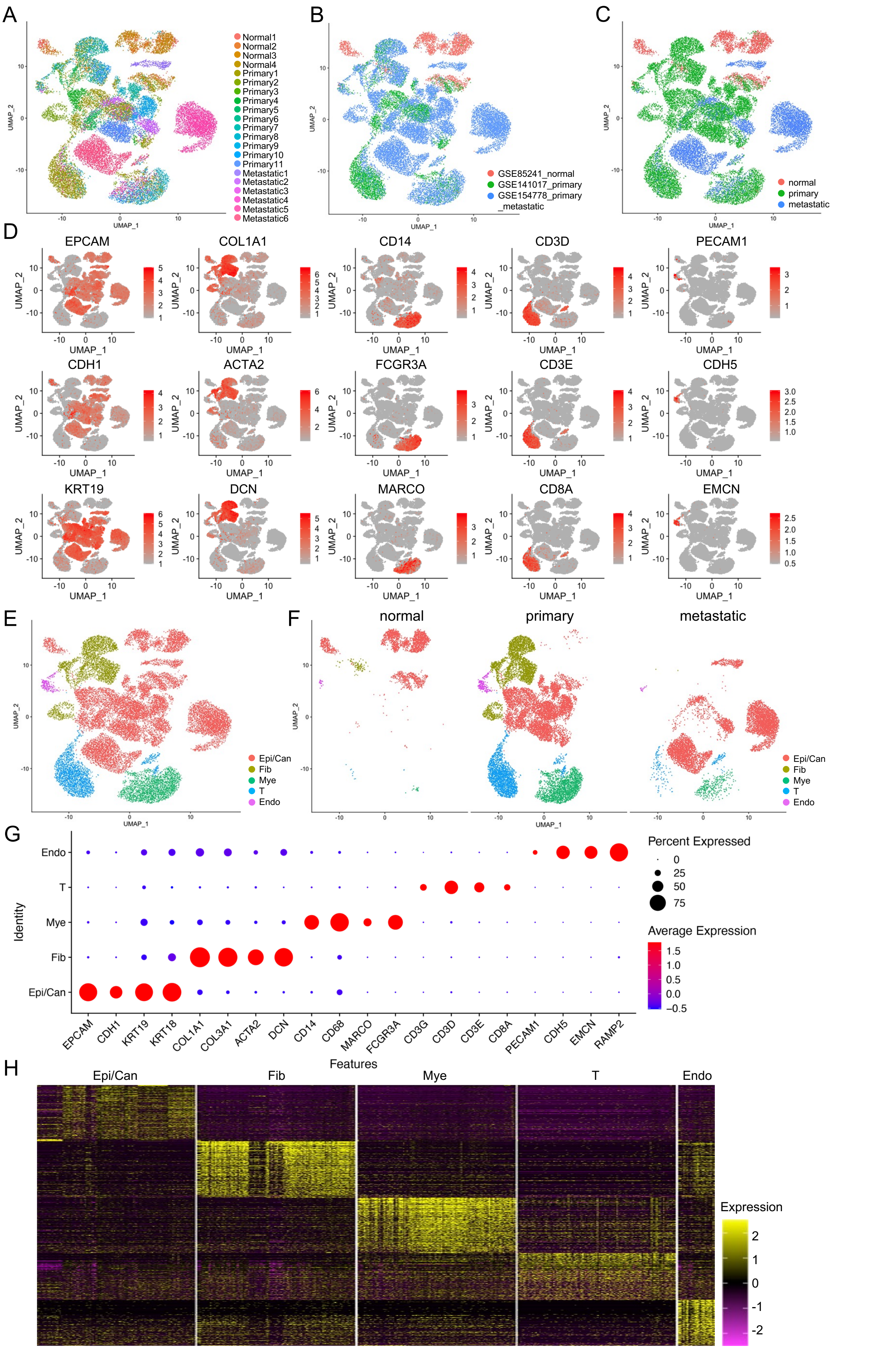

Supplemental Fig. 2 Integration of scRNA-seq data on primary pancreases, primary and metastatic PDAC tissues. Three single cell RNA-sequencing datasets were integrated, and visualization of each sample (**A**), each dataset (**B**) and each cancer status (**C**) by UMAP. (**D**) The expression of canonical cell type marker genes used for cell type identification. (**E**) Cell type definition based on the expression of cell type marker genes. (**F**) Split version of the cell type identification. (**G**) Dot plots showed the expression of the cell type marker genes in the defined cell types. (**H**) Heatmap of the expression of top 500 genes of each defined cell type in (**E**) was generated by "DoHeatmap" function in R-studio (Version 1.4.1717).

Figure S3

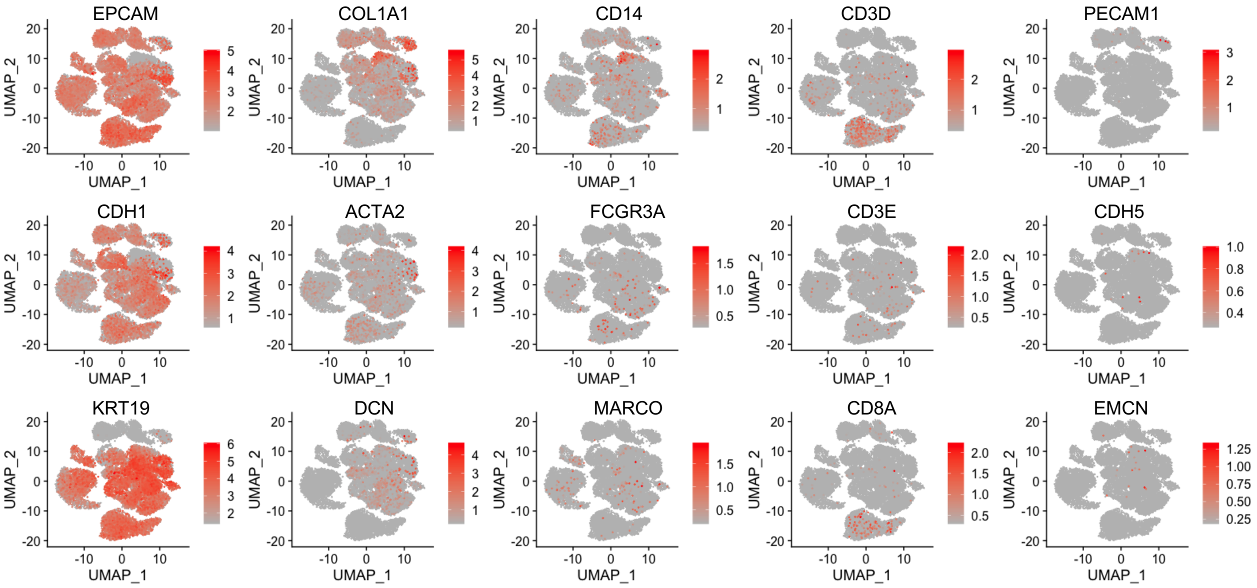

Supplemental Fig. 3 Purity of the epithelial cell lineage extracted were confirmed by the expression of canonical cell type markers.

Figure S4

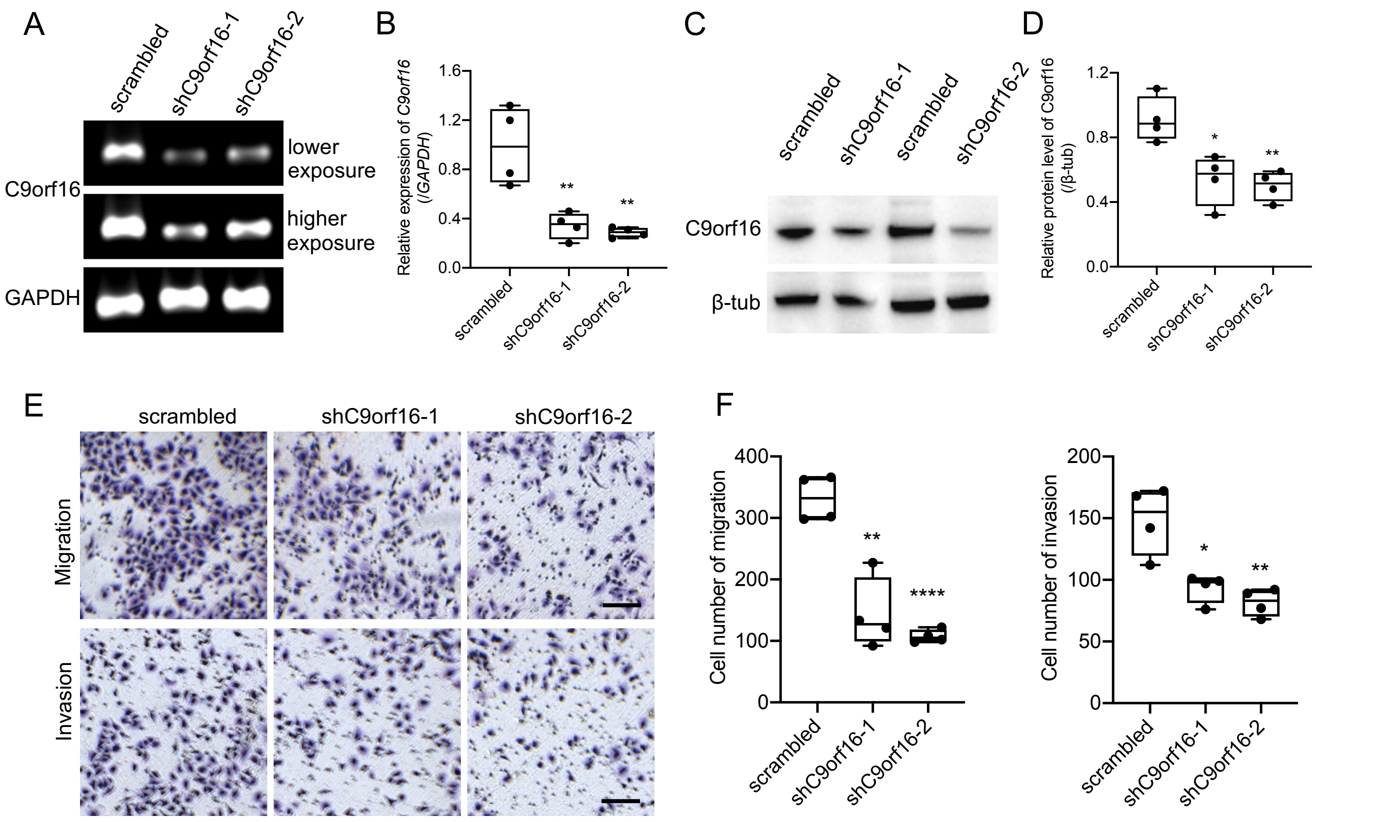

Supplemental Fig. 4 C9orf16 knockdown in BxPC-3 cells inhibited cancer cell invasion. RT-PCR (**A**), real time PCR (**B**) and western blotting (**C-D**) analysis were performed to confirm the knockdown efficiency of C9orf16 in BxPC-3 cells. Migration and invasion assays (**E**) and cell number quantification (**F**) of the C9orf16 knockdown and the scrambled BxPC-3 cells. Scale bar: 100 μm. **: $p < 0.01$; ***: $p < 0.001$; ****: $p < 0.0001$.

Figure S5

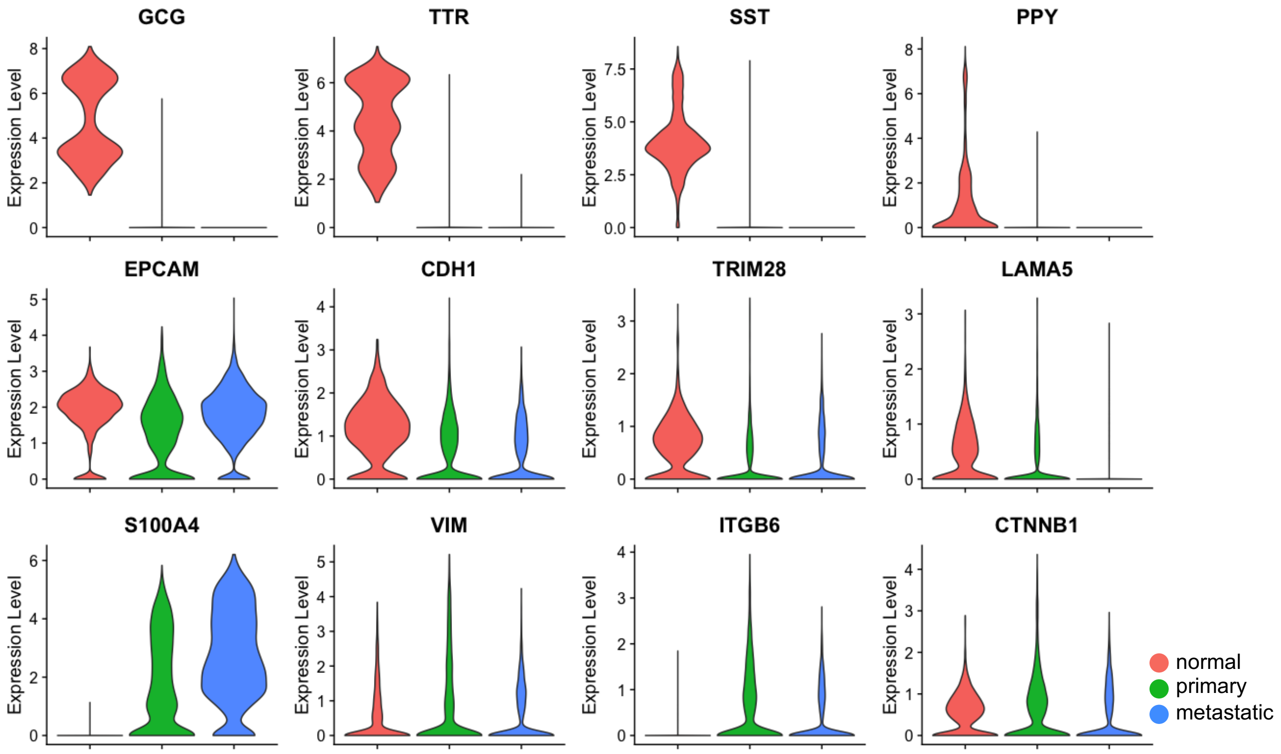

Supplemental Fig. 5 Violin plots showed the expression of EMT related genes.

Figure 4A

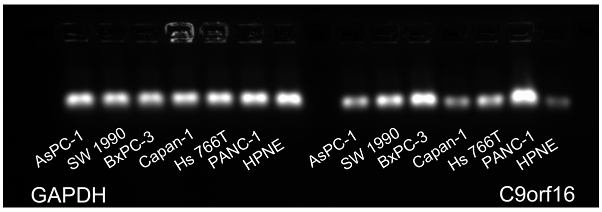

Figure 4B

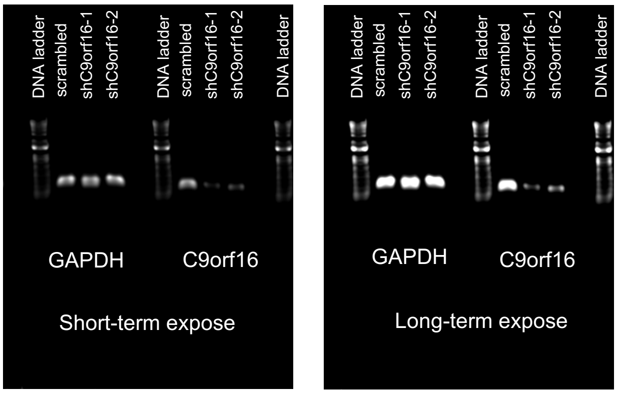

Figure 4D

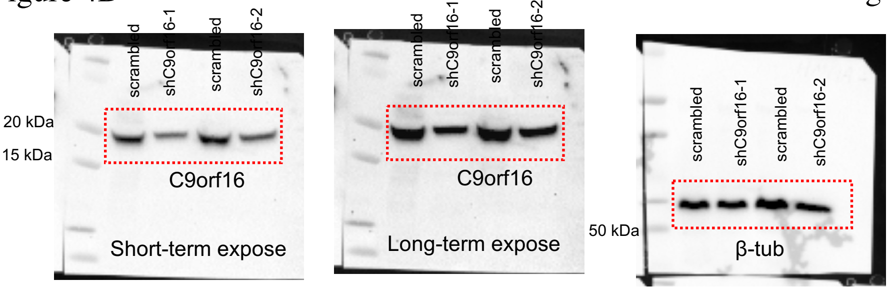

Figure 5A

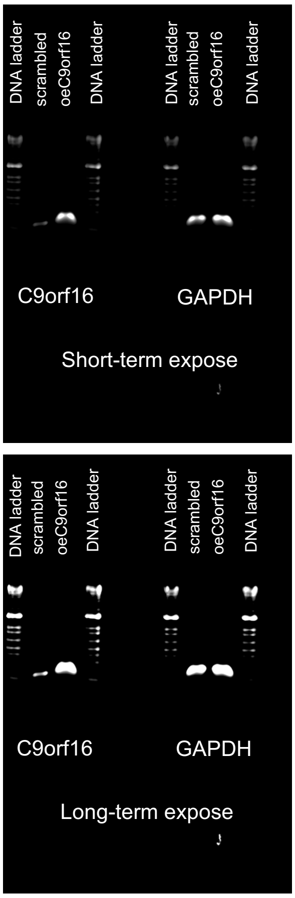

Figure 5C

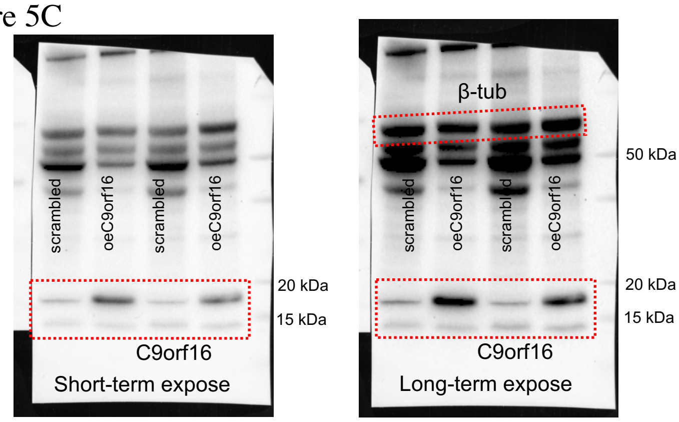

Figure 8C

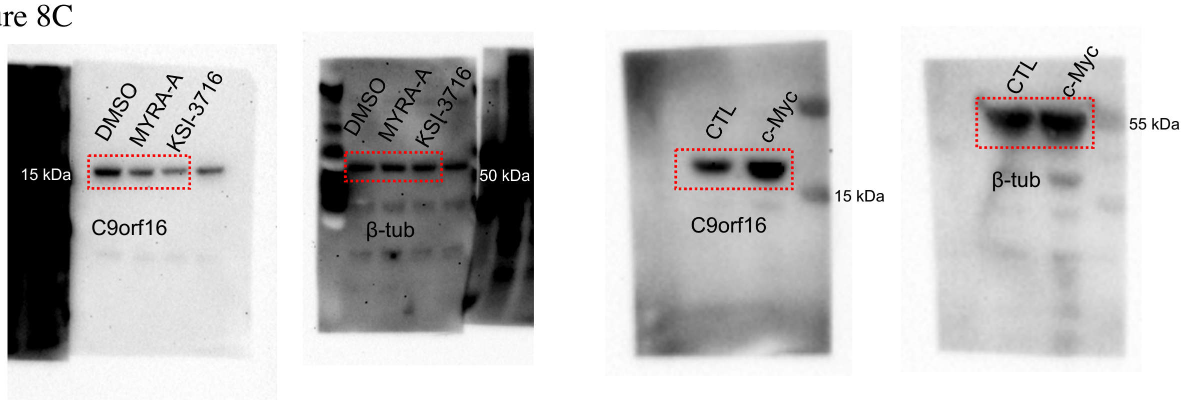

Supplemental Fig. 6 Original full-length gels and blots for each figure panel.

Supplemental Fig. 4A

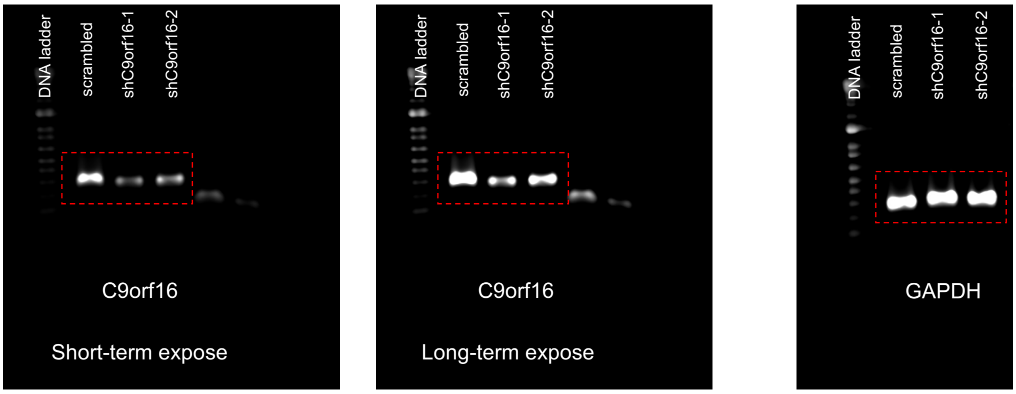

Supplemental Fig. 4C

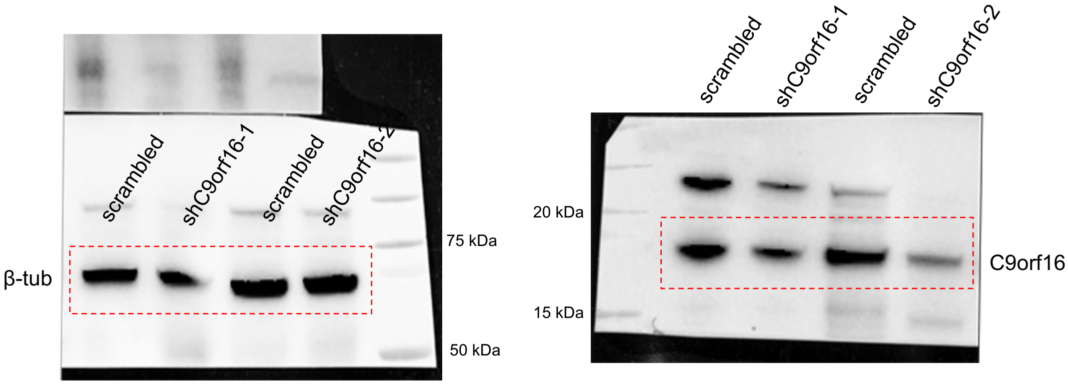

Supplemental Fig. 7 Original full-length gels and blots for each panel of Supplemental Fig. 4 .
